# Preparation and Evaluation of New Glycopeptides Obtained by Proteolysis from Corn Gluten Meal Followed by Transglutaminase-Induced Glycosylation with Glucosamine

**DOI:** 10.3390/foods9050555

**Published:** 2020-05-01

**Authors:** Xiao-Lan Liu, Chun-Li Song, Jia-Peng Chen, Xiang Liu, Jian Ren, Xi-Qun Zheng

**Affiliations:** 1 Key Laboratory of Corn Deep Processing Theory and Technology of Heilongjiang Province, College of Food and Bioengineering, Qiqihar University, Qiqihar 161006, China; liuxiaolan001@126.com (X.-L.L.); songchunli@qqhru.edu.cn (C.-L.S.); xdldpgy@163.com (J.-P.C.); liuxiang2018001@163.com (X.L.); renjian1970789@163.com (J.R.); 2 College of Food, Heilongjiang Bayi Agricultural University, Daqing 163319, China

**Keywords:** corn gluten meal, glycopeptides, property modification

## Abstract

New glycopeptides were generated by proteolysis from corn gluten meal (CGM) followed by transglutaminase (TGase)-induced glycosylation with glucosamine (GlcN). The glycopeptides exhibited desirable antioxidant and intracellular ROS-scavenging properties. The amount of conjugated GlcN quantified by high-performance liquid chromatography (HPLC) was 23.0 g/kg protein. The formed glycopeptides contained both glycosylated and glycation types, as demonstrated by the electrospray ionization time-of-flight mass spectrometry (ESI-TOF MS/MS). The glycopeptides exhibited scavenging capabilities against free radical diphenylpicrylhydrazyl (DPPH) and hydroxyl radicals by reducing their power. The potential protection of glycopeptides against ethanol-induced injury in LO2 cells was assessed In Vitro based on methyl thiazole tetrazolium (MTT) testing and intracellular reactive oxygen species (ROS) scavenging capacity, respectively. Glycopeptide cytoprotection was expressed in a dose-dependent manner, with the glycopeptides exhibiting good solubility ranging from 74.8% to 83.2% throughout a pH range of 2–10. Correspondingly, the glycopeptides showed good emulsifying activity (36.0 m^2^/g protein), emulsion stability (74.9%), and low surface hydrophobicity (16.3). These results indicate that glycosylation of CGM significantly improved its biological and functional properties. Glycopeptides from CGM could be used as potential antioxidants as well as comprising a functional food ingredient.

## 1. Introduction

In 2019, China’s yield of corn, one of the major cereal foods in the world, reached 260.77 million tons [1]. Approximately 14% of corn production is used to produce corn starch via a wet milling process [2], resulting in corn gluten meal (CGM) as the dominant co-product. CGM contains at least 60% (*w/w*) protein, including alcohol-soluble zein (68%) and alkali-soluble glutelin (27%) [3]. Zein and corn glutelin have unique amino acid compositions. Zein contains a high proportion of glutamic acid (21–26%), leucine (20%), proline (10%) and alanine (10%) [4], whereas corn glutelin is particularly rich in glutamic acid/glutamine (about 30%). Leucine and proline play extremely important roles by exerting antioxidant effects, while glutamine promotes gastrointestinal tissue regeneration following toxic injury [5]. However, CGM has low solubility in aqueous systems, thereby limiting its application in the food industry, with almost all of CGM applied as feedstuff. Therefore, finding a solution to efficiently improve the solubility of CGM, reveal its activities, and broaden its applications in the food industry is a meaningful venture.

The glycosylation of proteins exhibits the potential to improve its biological and functional properties. Transglutaminase (TGase)-mediated (EC 2.3.2.13) glycosylation is a promising method, although traditional Maillard glycation has been widely used. TGase catalyzes the conjugation between saccharides and proteins (i.e., glycosylation), where proteins are used as acyl group donators and saccharides containing primary amines act as acyl group acceptors [6]. The conjugation of proteins to hydroxyl groups on saccharide molecules increases protein hydratability, correspondingly improving its solubility and allowing the biological properties of saccharides to be simultaneously incorporated into the protein. The improvement of pea legumin and wheat gliadin solubility at their isoelectric points was achieved via TGase-induced glycosylation [7]. The immunomodulation and inhibition of *Escherichia coli* via TGase-induced glycosylated caseinate hydrolysates [8] and cold water fish skin gelatin [9] were also observed, respectively. 

The cross-linking of proteins and conjugation of saccharides occur simultaneously in TGase-induced glycosylation. TGase catalyzes the intermolecular and intramolecular protein cross-linking reactions between lysine and glutamine via acyl transfer, a process widely applied in the meat and dairy industries to improve gelation and protein texture [10], as well as varying the viscoelastic and rheological properties of the obtained products [11]. Controlling protein cross-linking is beneficial to saccharide conjugation. On one hand, reduced viscosity of the reaction system could benefit the reaction; however, a greater number of available reactive sites also increases saccharide conjugation. In this respect, CGM is a prospective substrate for TGase-induced glysosylation. The notable absence of lysine and the abundance of glutamine account for some intermolecular and intramolecular cross-linking reactions these molecules; correspondingly, TGase dominantly catalyzes glycosylation between saccharides and glutamine molecules. 

The objective of this study was to produce glycopeptides from CGM and evaluate their biological activities. The moderate proteolysis of CGM was first conducted as a pretreatment to obtain intensive reaction sites, with the following glycosylation of CGM hydrolysates in the presence of TGase and glucosamine (GlcN) to prepare new glycopeptides. Electrospray ionization time-of-flight mass spectrometry (ESI-TOF MS/MS) was used to confirm the occurrence of the glycosylation/glycation reaction and intracellular ROS production and solubility was evaluated alongside the emulsification capabilities of the glycopeptides.

## 2. Materials and Methods

### 2.1. Materials and Chemicals

CGM was provided by Longfeng Corn Development Co., Ltd. (Suihua, Heilongjiang, China), with a total protein content of 61.3%. GlcN was purchased from Sinopharm Chemical Reagent Co., Ltd. (Shanghai, China). TGase was purchased from Jiangsu Yiming Fine Chemical Industry Co., Ltd. (Qinxing, Jiangsu, China), with an activity of 1000 units (U) per gram (China). Alcalase (6.28 × 10^5^ U/mL) was a kind gift from Novo Nordisk (Bagsvaerd, Denmark). Pepsin (P-7000) and trypsin (T-7409) were purchased from Sigma. All other chemicals used were of analytical grade. 

### 2.2. CGM Purification 

CGM was purified from original CGM as per our previously reported method [12]. Briefly, CGM suspensions of 10% (*w/v*) in pH 6.0 phosphate buffer were incubated with α-amylase (30 U/g of protein) at 60 °C for 2 h. After boiling the solution to inactivate the enzyme, the mixture was filtered to remove starch hydrolysates. The residue was then washed with the same amount of water three times and centrifuged at 4500 r/min for 10 min. The precipitate was collected and dried to obtain the starch-removed CGM. The obtained CGM powder was mixed with acetone at a ratio of 10:1 (*v/w*) and stirred for 45 min. The mixture was then centrifuged at 4500 r/min for 10 min to collect the sediment, which was further processed twice using the same method and vacuum freeze-dried. The purified CGM was used to prepare the CGM hydrolysates.

### 2.3. Preparation of CGM Hydrolysates

CGM dispersions (5%, *w/v*) were hydrolyzed by Alcalase at an enzyme to substrate (E:S) ratio of 0.5% (*w/w*), under gentle stirring at 60 °C and pH 7.0. The reaction pH was maintained by adding 2.0 mol/L NaOH. The degree of hydrolysis (DH) of the CGM was determined by Adler–Nissen′s method [13]. After hydrolysis, the reaction was terminated by boiling for 15 min. The obtained hydrolysates were centrifuged at 4000 r/min for 10 min, and the supernatant was collected, lyophilized and used for further analysis.

### 2.4. Preparation of GlcN–CGM Hydrolysates

The lyophilized CGM hydrolysates and GlcN were dissolved separately and mixed. TGase was then added to the above mixtures and incubated at a specific temperature and pH value in a water bath with a constant agitation. The content of conjugated GlcN was measured as an index of the reaction conditions. The following parameters were used in this reaction: pH at 7.0, 7.5 and 8.0; temperature at intervals of 5 from 35 to 55 °C; CGM hydrolysate concentrations of 3%, 4% and 4.4% (*w/v*); mole ratio of CGM hydrolysates and GlcN of 1:2, 1:3 and 1:4; E:S ratio at intervals of 5 U/g from 5 to 20 U/g protein; and reaction time at intervals of 0.5 h from 1 to 4 h. The reaction was terminated by heating at 85 °C for 10 min. The obtained products were conducted with dialysis treatment against distilled water and the retentates (GlcN–CGM hydrolysates) were collected, lyophilized and stored at −18 °C. Cross-linked CGM hydrolysates were also prepared under the same conditions without the addition of GlcN. CGM, CGM hydrolysates and cross-linked CGM hydrolysates were used as controls.

In addition, in order to minimize any interference in the ESI-TOF MS/MS analysis, pure TGase from guinea pig liver tissue (Sigma-Aldrich) was used during preparation.

### 2.5. HPLC Analyses 

Conjugated GlcN was released from the prepared GlcN–CGM hydrolysates by HCl hydrolysis [14], and the glucosamine was reacted with anthranilic acid-derivatizing reagent. The stable derivative was then measured via the ultraviolet detection of HPLC analysis [15]. The analysis was performed on a Hitachi HPLC 2130 (Tokyo, Japan), equipped with an L-2400 UV-detector with an applied detection wavelength of 230 nm. A C_18_ column (50 mm × 4.6 mm, Waters Corporation, Milford, MA, USA) was used to analyze the glucosamine derivative with a gradient elution program. Solvent A consisted of 0.4% *n*-butylamine, 0.5% phosphoric acid and 1.0% tetrahydrofuran in water. Solvent B consisted of equal parts solvent A and acetonitrile. The elution was performed at 4% B for 30 min, followed by a linear increase to 100% B at 45 min [15]. The amount of conjugated GlcN was calculated using a regression equation obtained from a GlcN standard solution, expressed as g/kg protein.

### 2.6. Fourier-Transform Infrared (FTIR) Spectra 

For FTIR analysis, the samples (CGM hydrolysate, cross-linked CGM hydrolysate and GlcN–CGM hydrolysate) were mixed with KBr at a ratio of 1:50 (*w/w*), with 40 mg of the mixture prepared in KBr discs under dry air at room temperature. All FTIR spectra were obtained using a Spectrum One FTIR spectrometer (Perkin Elmer Inc., Norwalk, CT, USA) by scanning from 4000 to 400 cm^−1^ at a resolution of 1 cm^−1^. A total of 32 scans were used.

### 2.7. ESI-TOF MS/MS Analysis Conditions

#### 2.7.1. LC Chromatographic Conditions

The fraction separation of two CGM hydrolysates was carried out using an ACQUITY UPLC ® system (Waters, Milford, MA, USA) with a BEH (Ethylene Bridged Hybrid) column (particle size: 100 mm × 1.7 μm). The mobile phases A and B were acetonitrile and 0.1% formic acid in water, respectively. The flow gradient was 0–10 min, 15–50% A, curve 6, and 10–12 min, 50–100% A, curve 1 with a flow rate of 1 mL/min.

#### 2.7.2. Mass Conditions

The RP-HPLC system was connected to a Waters XEVO G2 Q-Tof MS equipped with an ESI interface. The desolvation temperature and desolvation gas flow of the ESI interface were 300 °C and 600 L/h, respectively. Positive ion intensities ranging between 100–1200 *m/z* were recorded using full-scan MS. Leucine–enkephalin was used as the mass spectrometry standard.

### 2.8. Evaluation of Antioxidant Properties of Glcn–CGM Hydrolysates and their In Vitro Digestive Products

The antioxidant properties of four CGM samples (CGM, CGM hydrolysates, cross-linked CGM hydrolysates and GlcN-CGM hydrolysates samples) and their In Vitro digestive products (pepsin and pepsin–trypsin digestions) were determined using our previously reported methods [16] with minor modifications. Four different methods (In Vitro), namely diphenylpicrylhydrazyl (DPPH) radical-scavenging ability, reducing power, hydroxyl radical-scavenging ability and Fe^2+^-chelating activity, were used to analyze the antioxidant properties of the samples. 

Four CGM samples were hydrolyzed by pepsin and pepsin–trypsin to indicate In Vitro digestibility according to the methods of Marciniak-Darmochwal and Kostyra [17] and Tang, Sun, Yin and Ma [18], respectively. Briefly, for one-step hydrolysis, 2 mg of pepsin was added to 10 mL of protein dispersion (1%, *w/v*, pH 2.0) and incubated at 37 °C for 2 h. The supernatants were collected by centrifugation at 10,000× *g* for 20 min. For two-step hydrolysis, 10 mL of protein dispersion (1%, *w/v*, pH 2.0) was subjected to incubation for 1 h at 37 °C with pepsin, as mentioned above. The mixture was then heated at 90 °C for 5 min and the sample was lyophilized. The reconstituted solution (10 mL, pH 8.0) was subjected to trypsin hydrolysis (6 mg) at 37 °C for 1 h. Finally, the supernatants were collected by centrifugation at 10,000× *g* for 20 min and used to analyze the antioxidant properties of the samples.

### 2.9. MTT Method for Antitoxicity Assay

Methyl thiazole tetrazolium (MTT) testing was used to evaluate the CGM peptide samples’ cytotoxicity in LO2 cells based on the work by Choe et al. [19]. LO2 cell mixtures were seeded into 96-well plates with a density of 1 × 10^5^ cells per well and allowed to attach at 37 °C for 6 h. Thereafter, the cells were cultured in the presence of various concentrations of peptide samples (0.005–1.0 mg/mL) for another 24 h. The wells were then washed in phosphate buffer and then incubated with MTT at a final concentration of 0.5 mg/mL for an additional 4 h. The remaining MTT solution was then carefully removed. Finally, 150 μL of dimethyl sulphoxide (DMSO) was added to dissolve the formed formazan. The absorbance at 570 nm was monitored by an EnSpire microplate reader (Perkin Elmer, Waltham MA, USA). Control cells were used in each assay. The cell viability was calculated as Cell viability (%) = OD _test_/OD _control_ × 100.

### 2.10. Detection of Intracellular ROS Production

The effect of GlcN–CGM hydrolysate addition on intracellular ROS production was measured using a 2′,7′-dichlorofluorescein diacetate (DCFH-DA) probe as an indicator, following the method of Vieira, da Silva, Carmo and Ferreira [20]. LO2 cell samples of 100 uL were seeded into 96-well plates with a density of 1 × 10^5^ cells per mL when they reached the logarithmic growth phase and allowed to attach at 37 °C for 6 h. The cells were then incubated for 4 h with different concentrations of peptides (0.05–2 mg/mL). Following the pretreatment with peptides, the cells were washed twice with PBS and incubated with the same volume of 3% (*v/v*) ethanol for 24 h. After that, the cells were washed twice with PBS and incubated in the presence of DCFH-DA for 20 min, followed by another two washes with PBS and the careful removal of the extracellular probe (DCFH-DA). The cells trapped the fluorescent dye (DCF) inside, allowing for the monitoring of the fluorescence values using an EnSpire microplate reader (Perkin Elmer, Waltham MA, USA) by excitation at 485 nm and emission at 530 nm. Two controls, i.e., cells treated with medium or 3% (*v/v*) ethanol only, were also included. The 2′,7′-dichlorofluorescein (DCF) fluorescein intensity values at Ex/Em 485/538 nm were measured. ROS (%) was expressed relative to the maximum ROS levels of the negative control, i.e., the cells treated with medium only.

### 2.11. Solubility

Lyophilized samples were dispersed in various buffers, including citrate–phosphate at pH 2.8–7.0, barbital sodium chlorhydric acid buffer at pH 7.0–9.5, Na_2_CO_3_/NaHCO_3_ at pH 9.5–10.4 and Na_2_HPO_4_/NaOH at pH 11.8. The four CGM samples (2 mg/mL) were centrifuged (8000× *g*) for 20 min after overnight rehydration at 4 °C, and the supernatant was collected to determine the protein content. The nitrogen solubility was expressed as a percentage of the protein content of the supernatant according to the initial total protein content of the dispersion [21].

### 2.12. Emulsifying Property

The emulsifying activity index (EAI) and the emulsifying stability index (ESI) of the four CGM samples were determined using a turbidimetric method by Pearce and Kinsella [22]. Emulsions were prepared by mixing soybean oil with 1 mg/mL protein dispersion (25 mL:75 mL), followed by homogenization for 1 min using a Model DS-1 high-speed homogenizer (Shanghai Specimen and Model Factory, Shanghai, China). Samples containing 10 μL of the prepared emulsion were mixed adequately with 5 mL of phosphate buffer (0.1 mol/L, pH 7.0) containing 1 mg/mL sodium dodecyl sulphate. The absorbance of the emulsions was recorded at 500 nm using an UV 752 spectrophotometer (Xinmao Instrument Co. Ltd., Shanghai, China). The EAI and ESI were calculated as follows (Equations (1) and (2)):

(1) $$\mathrm{EAI}\;(m^{2}/g)=\frac{2\times 2.303\times A_{0}\times \text{dilution}}{C\times (1-\varphi )\times 10^{4}}$$

(2) $$\mathrm{ESI}\;(\%)=\frac{A_{10}}{A_{0}}\times 100$$

where C is the concentration of the aqueous phase protein (10 kg/L), φ is the volumetric fraction of oil and A_0_ and A_10_ are the absorbance values of the initial emulsion formation after the maintenance of a static condition for 10 min, respectively. 

### 2.13. Surface Hydrophobicity

Surface hydrophobicity was determined on the basis of the procedures described by Hayakawa and Nakai [23]. Four CGM samples dispersed in sodium phosphate buffer (10 mmol/L, pH 7.0) were centrifuged at 10,000× *g* for 15 min and the supernatants were collected. A total of 4 mL of obtained supernatant was mixed with 20 μL of 1-anilino-8-naphthalene-sulphonate (ANS, 8.0 mmol/L). The fluorescence intensities of the mixtures were recorded at 390 nm (excitation wavelength) and 470 nm (emission wavelength) at a range of protein concentrations, from 0.025 to 0.4 mg/mL. The initial slope of the fluorescence intensity versus the protein concentration plot was regressed and expressed as an index of the protein surface hydrophobicity. 

### 2.14. Statistical Analysis

All data were expressed as means or means ± standard deviations from three independent experiments. Differences between the means of multiple groups were analyzed via post-hoc testing of one-way analysis of variance (ANOVA), alongside Duncan′s multiple range tests. SPSS 13.0 software (SPSS Inc., Chicago, IL, USA) was used to analyze the data.

## 3. Results and Discussion

### 3.1. Establishment of Glycosylation Reaction Conditions 

In order to obtain intensive reaction sites during CGM glycosylation, the moderate proteolysis of CGM was adopted as a technical pretreatment step before TGase-induced CGM glycosylation. Insoluble CGM was first subjected to hydrolyzation by Alcalase at an E:S of 0.5 *w/w*. The soluble supernatant was collected when the DH of CGM reached 5% in order to obtain more reaction sites in the following TGase-induced glycosylation. 

To achieve a high degree of glycosylation, the reaction time, pH, temperature, E:S ratio, CGM hydrolysate concentration, and the mole ratios of the CGM hydrolysate and GlcN were optimized. The results show that the optimal pH was 7.5, which fell in the range provided by the manufacturer. The optimum reaction temperature, the ratio of E:S and CGM hydrolysate concentration were 45 °C, 10 U/g protein and 4% (*w/v*), respectively. As more intensive conjugation was observed at higher concentrations of acyl acceptors (GlcN), a technologically reasonable mole ratio of CGM hydrolysate to GlcN (1:3) was applied. Under the above conditions, the optimal reaction time was 3 h. Under the optimized conditions, the amount of GlcN in the GlcN–CGM hydrolysates, quantified by HPLC, was 23.0 g/kg. This value was higher than that of the glycosylated soybean protein (2.62 g/kg SPI) [24] and casein (10.3 g/kg casein) [25], which were catalyzed by TGase. The greater amount of glutamine in the CGM hydrolysate substrate provided more available reactive sites for GlcN conjugation during the reaction.

### 3.2. Evaluation of Glycoconjugation

#### 3.2.1. HPLC

HPLC was used to analyze the modified CGM hydrolysates to qualify the amount of conjugated GlcN. The retention times of the standard GlcN were 9.60 and 10.66 min, which, respectively, corresponded to AA–GlcN and its epimer (data not shown). As expected, the same phenomenon was also observed in the GlcN–CGM hydrolysates but not in CGM hydrolysates, potentially giving preliminary evidence of GlcN conjugation. 

#### 3.2.2. FTIR

**Figure 1:**
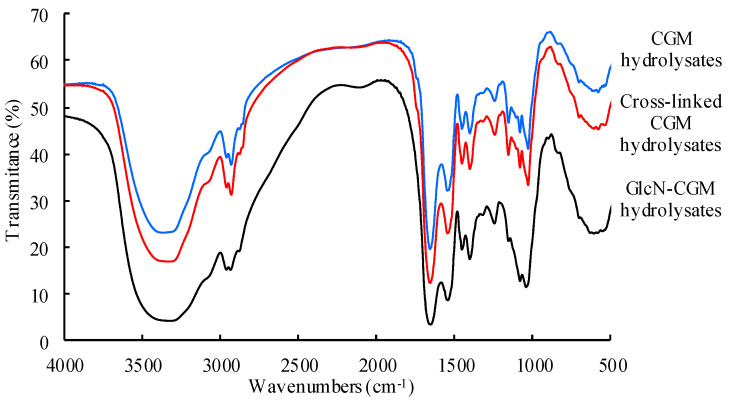
Fourier-transform infrared (FTIR) spectra in KBr pellets of corn gluten meal (CGM) hydrolysates, cross-linked CGM hydrolysates and glucosamine (GlcN)-CGM hydrolysates.

The FTIR absorbance spectra of the samples provide information about unique chemical bonds, so the FTIR analytical technique was used here to identify different components. FTIR spectroscopy was also used to show changes in the CGM hydrolysates side-chains after treatment with TGase and GlcN; these results are shown in Figure 1. Typical –C–O stretching and –OH deformation vibrations occurred at 1050–1150 cm^−1^. Intense deformation vibrations were observed when the hydroxyl groups of the saccharide molecules were conjugated into proteins. Compared with the CGM hydrolysates, the absorbance at 1025 cm^−1^ of the GlcN–CGM hydrolysates was significantly strengthened, as expected. These results indicate that the GlcN–CGM hydrolysates contained more –OH groups via covalent bonds, meaning that the modified products contained groups from GlcN. When free saccharides were removed, the glycopeptides were regarded as obtained via TGase-induced glycosylation. A similar phenomenon was also observed in soy protein isolate grafts obtained by glycation [26].

#### 3.2.3. ESI-TOF MS/MS

**Figure 2:**
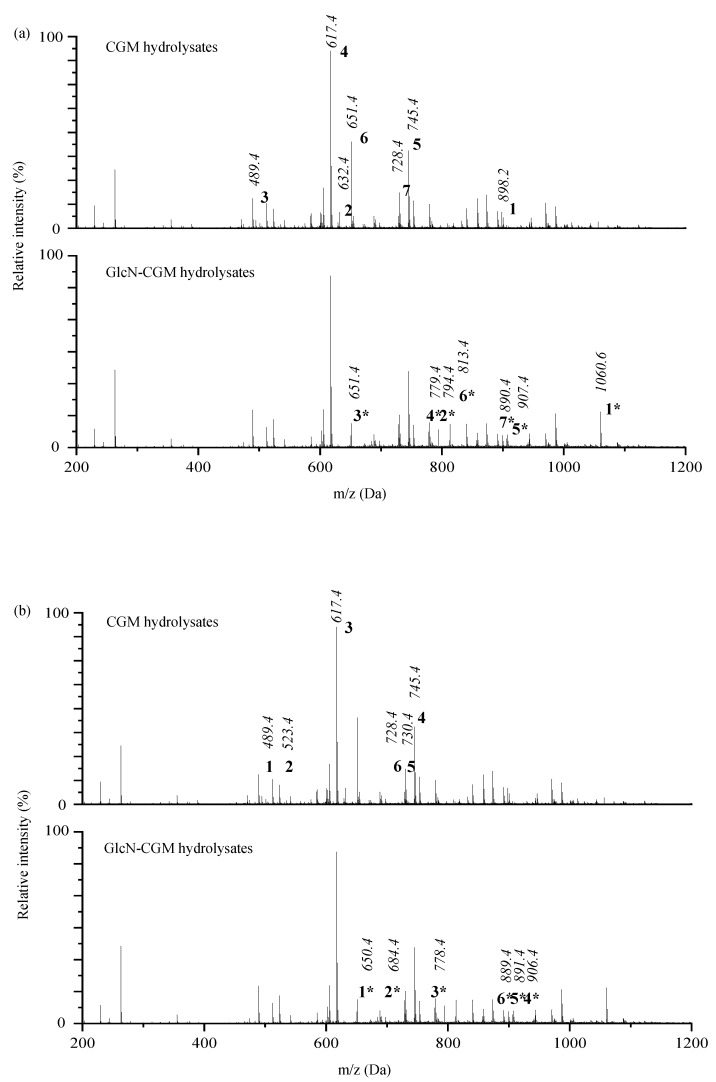
ESI-MS/MS spectra of CGM hydrolysates (**a**) and CGM hydrolysates conjugated with GlcN by TGase at 37 °C (**b**). GlcN-CGM hydrolysates and natural CGM hydrolysates are indicated with the same number.

Electrospray ionization mass spectrometry (ESI-MS/MS) analysis is a useful tool to qualitatively describe glycopeptides carrying N-acetyl glucosamine on a specific amino acid. Depending on the mechanism of the TGase-catalyzed glycosylation reaction, the primary amino groups of the glutamine residues in CGM hydrolysate chains function as acyl donors, whereas GlcN acts as an acyl acceptor. Conjugating GlcN to glutamine residues is accompanied by NH_3_ release. The presence of glycosylation in peptides (glycopeptides) was evaluated by a mass shift of 162 Da (GlcN: 179 Da; NH_3_: 17 Da) in comparison with the original peptide. Seven glycopeptides with high intensity were found from the GlcN–CGM hydrolysation (Figure 2a), with molecular weights of 651.4, 779.4, 794.4, 813.4, 890.4, 907.4 and 1060.6 Da. These results show that GlcN was conjugated into CGM hydrolysates. TGase-induced glycosylation of fish skin gelatin hydrolysates in the presence of GlcN was also confirmed by another MS method (MALDI-TOF-MS) [9].

Heating the mixture was used to deactivate the TGase during preparation. The possibility of traditional Maillard-type glycation between GlcN and CGM hydrolysates was considered. The mass shift of 161 Da (GlcN: 179 Da; H_2_O: 18 Da) was also taken into account. Six glycopeptides with the following molecular weights were produced through glycation: 650.4, 684.4, 778.4, 889.4, 891.4 and 906.4 Da (Figure 2b). ESI-MS/MS analysis indicated that GlcN was conjugated into CGM hydrolysates via TGase- and Maillard-type reactions based on our glycopeptide preparation process.

### 3.3. Biological Properties of Glycopeptides

#### 3.3.1. Antioxidant Properties In Vitro of the Prepared Glcn–CGM Hydrolysates and its Digestion Products

**Figure 3:**
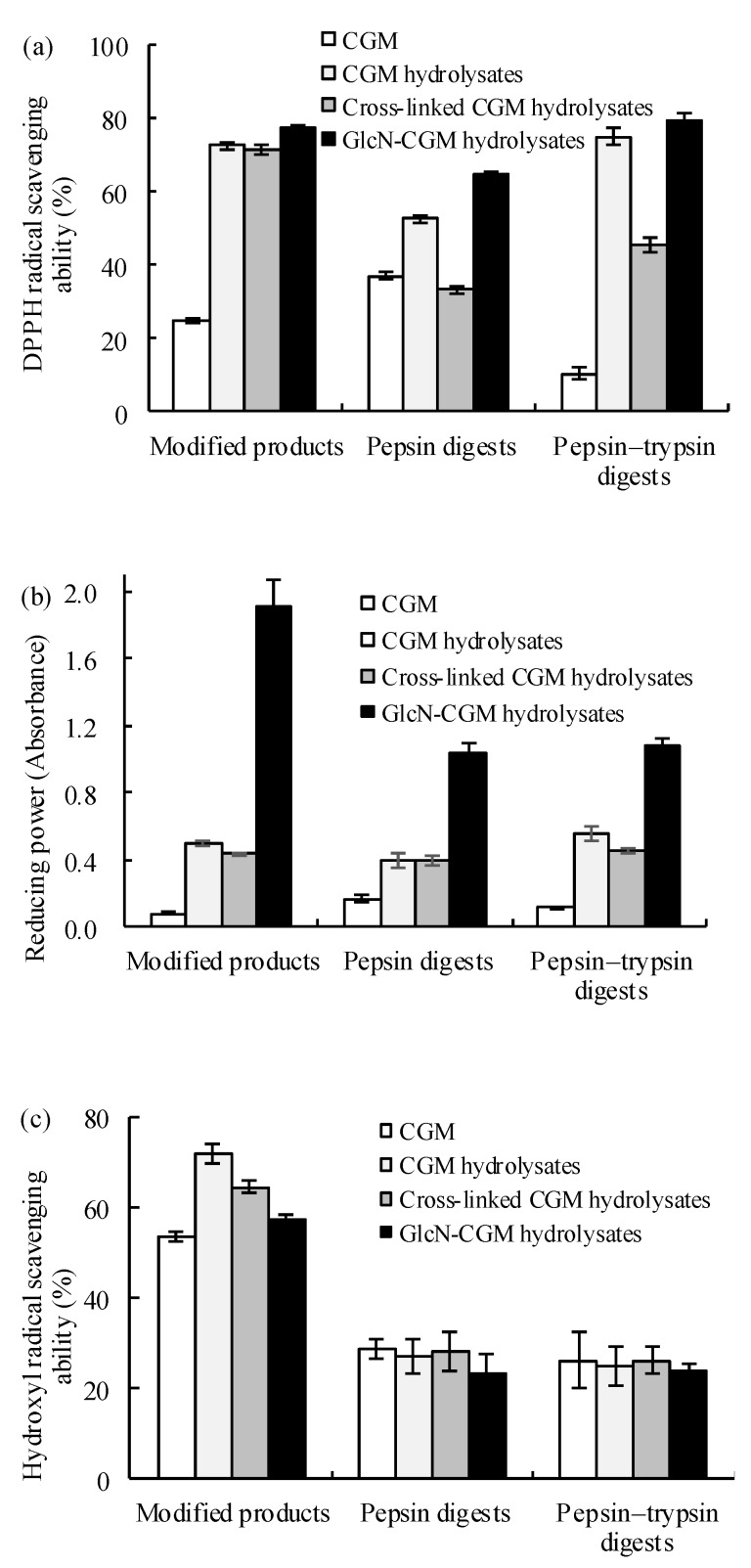

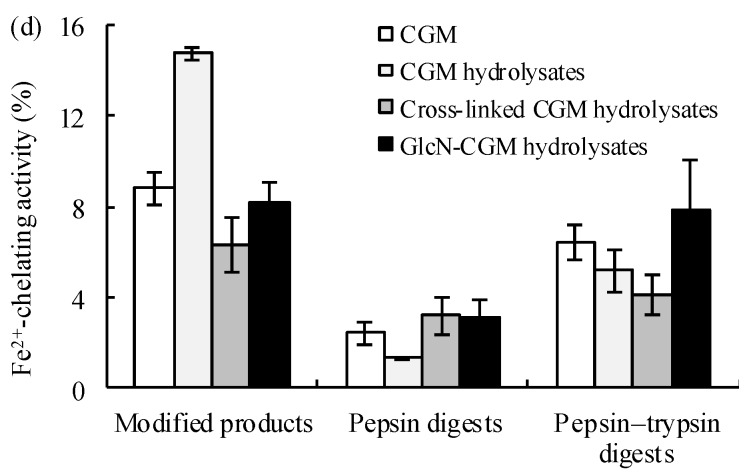
Antioxidant activity of CGM, CGM hydrolysates, cross-linked CGM hydrolysates, GlcN–CGM hydrolysates and their products after being subjected to pepsin and pepsin–trypsin digestions. (**a**) Diphenylpicrylhydrazyl (DPPH) radical-scavenging activity (2.0 mg/mL), (**b**) reducing power (5.0 mg/mL), (**c**) hydroxyl radical-scavenging activity (2.0 mg/mL) and (**d**) Fe^2+^-chelating activity (2.0 mg/mL) (*n* = 3, error bars show standard deviation).

DPPH is a typical method used to evaluate the antioxidant activities of compounds In Vitro, depending on a quantitative relationship between the degree of DPPH fading and the number of proton donators [27]. GlcN–CGM hydrolysates exhibited the highest scavenging activity against DPPH (77.5%) at 2 mg/mL, which was higher than the CGM hydrolysates (72.5%) and about three times greater than CGM (24.86%) (Figure 3a). The conjugated saccharide moiety and the hydrolysis treatment caused more electrons to be donated. These electrons reacted with free radicals, thereby preventing the radical chain reaction. Similar results were also reported regarding the radical-scavenging ability of DPPH due to the increase of glycated gluten hydrolysates after the treatment of GlcN by TGase [28]. High DPPH radical-scavenging activities were also observed when the CGM hydrolysates and their GlcN-conjugated products were hydrolyzed by pepsin or pepsin–trypsin (Figure 3a), suggesting that GlcN conjugation with CGM hydrolysates enhanced radical-scavenging activity.

Capacity reduction was used as an indicator of the antioxidant activities of chemicals based on the ability of some reducing substances in the sample to donate electrons or hydrogen ions, thereby resulting in the interruption of free radical chain reactions. The GlcN–CGM hydrolysates had an extremely high reducing capacity, which was significantly increased by about 23-fold and 2.8-fold in comparison with CGM and CGM hydrolysates (1.916 vs. 0.080 and 0.499) (Figure 3b). After In Vitro digestion, the digested GlcN–CGM hydrolysates still showed desirable reducing power, although this decreased after peptic or tryptic hydrolysis (Figure 3b). The enhanced reducing power of the GlcN-conjugated CGM hydrolysates could be explained by the conjugation of GlcN, which can donate electrons or hydrogen ions. It was reported that the glycosylation of CGM with chitosan improved its reducing power [29], thereby supporting our results. 

All of the CGM products exhibited good hydroxyl radical-scavenging activities, ranging from 53.5% to 71.7% (Figure 3c), which remained (23.1–28.7%) after being subjected to In Vitro digestion. 

The Fe^2+^-chelating activity of GlcN-conjugated CGM hydrolysates was 8.2% (Figure 3d). CGM and its hydrolysates exhibited higher Fe^2+^-chelating activity (8.8% vs. 14.7%). A coordination compound was formed between Fe^2+^ and CGM hydrolysates under Fe^2+^-chelation, where the hydrogen atoms of the CGM hydrolysate molecules were able to act as ligating atoms. When the saccharide groups were conjugated to the CGM hydrolysates to generate new compounds (GlcN-conjugated CGM hydrolysates), some hydrogen atoms were replaced by polyhydric saccharide groups, thereby reducing their coordinating capability. Consequently, the Fe^2+^-chelating activity of the GlcN-conjugated CGM hydrolysates decreased. However, the GlcN-CGM hydrolysates had nearly the same Fe^2+^-chelation activity after being subjected to pepsin and pepsin–trypsin digestions (Figure 3d). 

Improved overall antioxidant properties In Vitro in GlcN-CGM hydrolysates were observed, indicating that GlcN-CGM hydrolysates could potentially be used as antioxidant additives in the food industry and related fields. Evaluation of the effect of addition of GlcN-CGM hydrolysates on oxidative stress (intracellular ROS generation) at the cellular level is therefore a meaningful venture.

#### 3.3.2. Effect of GlcN–CGM Hydrolysate Addition on Cell Viability of LO2 Cells

**Figure 4:**
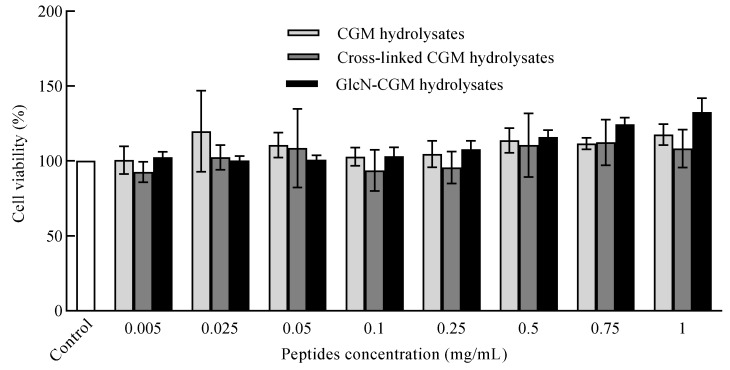
Cell viability assay of CGM hydrolysates, cross-linked CGM hydrolysates and GlcN–CGM hydrolysates at different concentrations in LO2 cells assayed via methyl thiazole tetrazolium (MTT). The values represent the mean ± SD of three independent experiments.

The analysis of cell viability, such as through toxicity assays, plays a vital role in all cell culture systems [30]. Cell viability is often defined as the number of healthy cells in a sample, which was used as the index in this experiment. MTT assays were applied to test the cytotoxicity of the GlcN–CGM hydrolysates; the resulting cell viability values are shown in Figure 4. The GlcN–CGM hydrolysates and CGM hydrolysates showed nearly the same or greater cell viability than the control after the cells were incubated at concentrations ranging from 0.05 to 1 mg/mL for 24 h. LO2 cell pretreatment with GlcN–CGM hydrolysates at a concentration range of 0.75–1 mg/mL resulted in very high cell viability (124.5–132.7%); therefore, GlcN–CGM hydrolysates and CGM hydrolysates, exhibited a protective effect on hepatocytes to some extent. Gelatin hydrolysates are capable of protecting hepatocytes, according to reports [31]. Moreover, Maillard reaction products form galactose and a gelatin hydrolysate exhibited low cytotoxicity in RAW264.7 cells in comparison with untreated cells [32]. 

#### 3.3.3. Effect of GlcN–CGM Hydrolysate Addition on Intracellular ROS Generation in LO2 Cells

ROS play important roles in cellular physiopathology, but excess ROS can impair proteins, lipids and DNA at the cellular level [33]. The detection of intracellular ROS generation is very important, especially when the redox balance of cells is disturbed. DCFH-DA was used as a membrane-permeable probe to detect intracellular ROS [34]. The effects of GlcN–CGM hydrolysates on oxidative stress in LO2 cells caused by exposure to ethanol were measured. 

**Figure 5:**
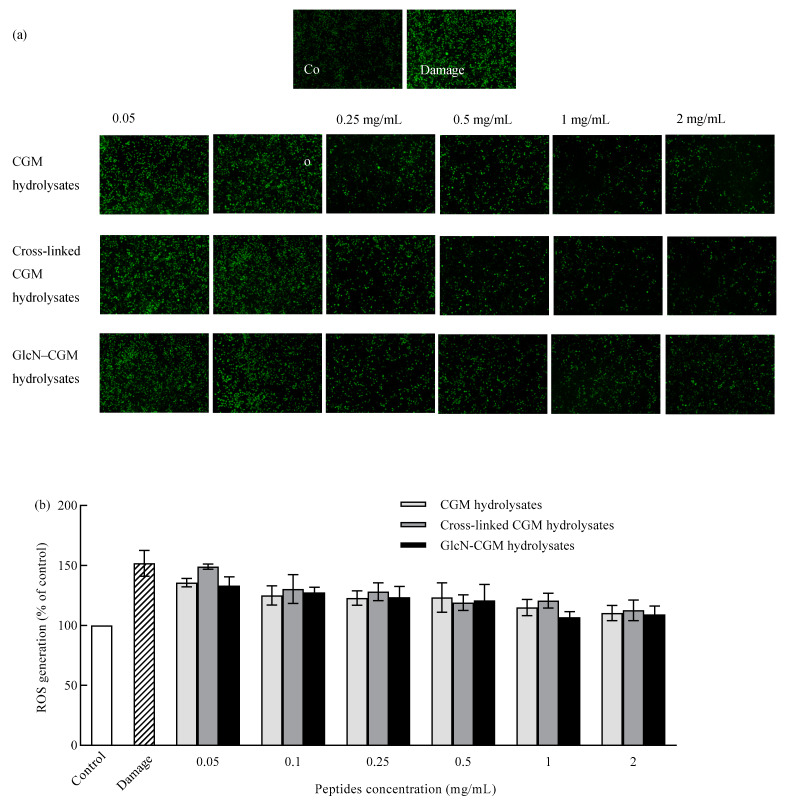
Intracellular reactive oxygen species (ROS) production in LO2 cells pretreated with CGM hydrolysates, cross-linked CGM hydrolysates or GlcN–CGM hydrolysates. (**a**) Photomicrographs of LO2 cells. Control: cells without any treatment; damage: cells treated with 3% (*v/v*) ethanol only. CGM hydrolysates, cross-linked CGM hydrolysates or GlcN–CGM hydrolysates were pretreated with the corresponding CGM hydrolysates samples for 4 h at 0.05 to 2 mg/mL, followed by 24 h ethanol exposure at 3% (*v/v*). The samples stained with 2′,7′-dichlorofluorescein diacetate (DCFH-DA). (**b**) Intracellular ROS production values are expressed as the percentage of fluorescence intensity relative to the control. The values are expressed as means ± SD of at least three independent experiments.

Florescent intensities of stained LO2 cells reflected using a micrograph were used to depict the production of the intracellular ROS of the samples; these results are shown in Figure 5a. Ethanol significantly induced oxidative stress to increase the intracellular ROS generation of LO2 cells and strong florescent intensity was observed. The addition of GlcN–CGM hydrolysates and CGM hydrolysates gradually decreased the intracellular fluorescence intensity and even suppressed it at a concentration of 1 mg/mL. Therefore, GlcN–CGM hydrolysates significantly ameliorated ethanol-induced oxidative damage by reducing intracellular ROS generation. Specifically, the high DCFH-DA fluorescent intensity decreased in a dose-dependent manner with the treatment of GlcN–CGM hydrolysates and CGM hydrolysates (Figure 5b). Markedly decreased intracellular ROS levels of 102.1% and 107.8% were observed in GlcN-CGM hydrolysate and CGM hydrolysate experiments under a concentration of 1 mg/mL for 4 h after 3% (*v/v*) ethanol-induced injury to LO2 cells, which was similar to the control group (*p* > 0.05) (Figure 5b). These results indicate that CGM hydrolysates exhibit protection toward cells, which was consistent with our MTT test results. The added products could alter the environment of the cells [35]; therefore, the ROS production required to oxidize intracellular DCFH to fluorescent DCF in GlcN–CGM hydrolysates was decreased. 

### 3.4. Functional Properties of Glycopetides

#### 3.4.1. Solubility

**Figure 6:**
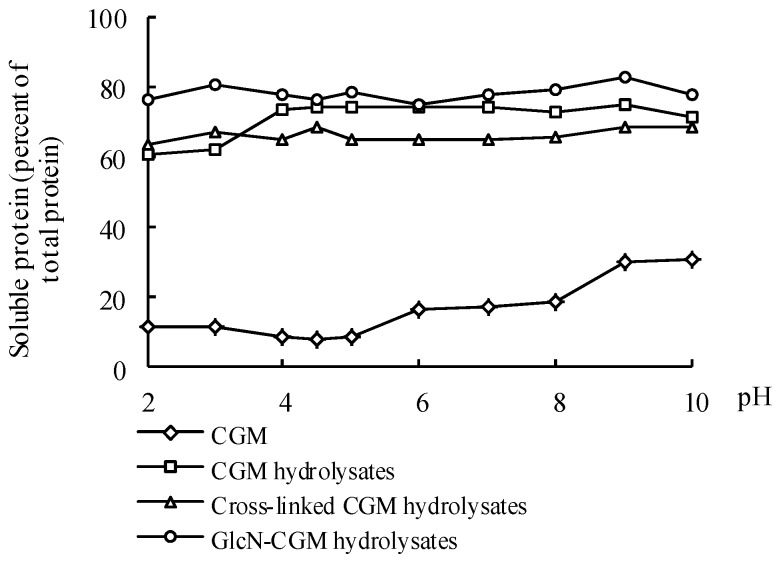
Solubility–pH profiles of CGM, CGM hydrolysates, cross-linked CGM hydrolysates and GlcN-CGM hydrolysates.

Solubility is a critical criterion for protein functionality [36] and may affect other functional properties, such as emulsification and foaming [37]. The solubility curves of CGM, CGM hydrolysates, cross-linked CGM hydrolysates and GlcN-CGM hydrolysates throughout a pH range of 2–11 are shown in Figure 6. GlcN–CGM hydrolysate solubility majorly increased (74.8–83.2%) by a much greater proportion than that of CGM (8.3–31.2%). CGM showed the lowest solubility of all the tested compounds throughout the pH range, presumably due to its possession of large patches of hydrophobic surface residues [38]. The enhanced solubility of the GlcN–CGM hydrolysates was mainly due to the hydrolysis treatment of the CGM, allowing for improved solubility of the CGM hydrolysates (60.5–75.0%) (Figure 6). In addition, the increased hydroxyl groups provided by the attached saccharides were also partly attributed to the improved solubility of the GlcN–CGM hydrolysates. Moreover, the attached saccharides buried some hydrophobic residues in the interior of the protein molecules. Wang et al. reported that the TGase-catalyzed glycosylation of CGM with GlcN increased its solubility [29]. The high solubility of the GlcN–CGM hydrolysates was not dependent on pH value, which is a property that could increase their application benefits in the food industry. 

#### 3.4.2. Emulsifying Properties

Protein emulsification is one of the most important interfacial properties of glycoproteins. The turbidimetric technique is often applied to evaluate emulsification. The emulsifying properties of GlcN–CGM hydrolysates, expressed as EAI and ESI, are shown in Table 1. GlcN–CGM hydrolysates and cross-linked CGM hydrolysates (36.0 and 57.1 m^2^/g protein) exhibited significantly (*p* < 0.05) higher EAI values than CGM and CGM hydrolysates (28.2 and 28.1 m^2^/g protein, respectively). Meanwhile, the emulsions stabilized by the GlcN–CGM hydrolysates and the cross-linked CGM hydrolysates (74.9 and 70.7%) showed much higher ESI than the CGM hydrolysates (63.4%). The GlcN-conjugated CGM-soybean oil system exhibited higher EAI and ESI values under a pH of 7, demonstrating that the oil was able to effectively disperse into the GlcN–CGM hydrolysate solutions while maintaining the stability of the layers between the proteins and lipids. Saccharide (GlcN) conjugation and cross-linking of CGM hydrolysates exhibited positive effects regarding emulsification improvement. On one hand, saccharide groups that adsorbed on the interfacial surface declined the oil–water separation rate, and saccharide (GlcN) conjugation with CGM hydrolysate molecules improved emulsion stability. On the other hand, TGase-mediated cross-linking of proteins gave rise to an increase in negative charges by blocking lysine residues, thereby exhibiting a synergistic effect and enhancing emulsion stability [39]. 

#### 3.4.3. Surface Hydrophobicity

Surface hydrophobicity is also a vital functional property regarding the stabilization of food protein intermolecular structures. The more distributed the aromatic and aliphatic amino acid residues on protein surfaces are, the greater the surface hydrophobicity. The surface hydrophobicity of the GlcN–CGM hydrolysates is shown in Table 1. The GlcN–CGM hydrolysates presented a lower surface hydrophobicity index (16.3) than the CGM hydrolysates and cross-linked CGM hydrolysates (28.1 and 20.4), but a value higher than CGM (8.1), indicating that ANS probes did not easily bind the hydrophobic regions of CGM. A high surface hydrophobicity in the CGM hydrolysates was observed because a flexible exposure high ratio of the hydrophobic amino acids surface zones (Ala of 6.89%, Val of 5.70%, Leu of 11.39%, etc.) occurred during hydrolysis. However, GlcN conjugation and the cross-linking of CGM hydrolysates decreased surface hydrophobicity. TGase catalyzed the glycosylation between the saccharide moieties and β-lactoglobulin [40], or the cross-linking of whey proteins [41] and a decrease in overall surface hydrophobicity. 

## 4. Conclusions

New GlcN-conjugated CGM hydrolysates (glycopeptides) were successfully produced by TGase-mediated glycosylation between CGM hydrolysates and GlcN. The glycosylation of CGM could enhance its antioxidant activities and reveal functional properties that are not found in the natural form. The strategy adopted in the present study could open up new economic opportunities for CGM utilization, allowing new glycopeptides from CGM to be applied as additives to certain functional foods.

## Figures and Tables

**Table 1:** Four evaluated indices of CGM, CGM hydrolysates, cross-linked CGM hydrolysates and GlcN-CGM hydrolysates (mean ± standard deviation, *n* = 3).

Functional Property	CGM	CGM Hydrolysates	Cross-linked CGM Hydrolysates	GlcN-CGM Hydrolysates
Glucosamine (g/kg protein)	0	0	0	23.0 ± 0.11
Surface hydrophobicity	8.1 ± 0.45 ^a^	28.1 ± 0.85 ^d^	20.4 ± 1.49 ^c^	16.3 ± 0.77 ^b^
Emulsifying activity index (m^2^/g protein)	28.2 ± 1.2 ^a^	28.1 ± 2.2 ^a^	57.1 ± 3.4 ^c^	36.0 ± 2.3 ^b^
Emulsion stability index (%)	39.0 ± 2.2 ^a^	63.4 ± 4.2 ^b^	70.7±2.8 ^c^	74.9 ± 1.9 ^d^

Different lowercase letters represent the superscripts after the values in same row indicate that one-way ANOVA of the means is significantly different (*p* < 0.05).

## Abbreviations

CGM	Corn gluten meal
GlcN	Glucosamine
TGase	Transglutaminase
HPLC	High performance liquid chromatography
FTIR	Fourier transform infrared spectroscopy
ESI-TOF MS/MS	Electrospray ionisation-time-of-flight mass spectrometry
